# Peak-Inspiratory-Flow-Rate Guided Inhalation Therapy Reduce Severe Exacerbation of COPD

**DOI:** 10.3389/fphar.2021.704316

**Published:** 2021-06-29

**Authors:** Shih-Yu Chen, Chun-Kai Huang, Hui-Chuan Peng, Hsing-Chen Tsai, Szu-Ying Huang, Chong-Jen Yu, Jung-Yien Chien

**Affiliations:** ^1^ Department of Internal Medicine, National Taiwan University Hospital Hsin-Chu Branch, Hsinchu, Taiwan; ^2^ Department of Internal Medicine, National Taiwan University Hospital, National Taiwan University College of Medicine, Taipei, Taiwan; ^3^ Institute of Epidemiology and Preventive Medicine, College of Public Health, National Taiwan University, Taipei, Taiwan; ^4^ Department of Nursing, National Taiwan University Hospital, National Taiwan University College of Medicine, Taipei, Taiwan; ^5^ Department of Pharmacy, Taipei City Hospital Songde Branch, Taipei, Taiwan

**Keywords:** peak inspiratory flow rate, chronic obstructive pulmonary disease, inhalers and drugs, drug delivery systems, exacerbation

## Abstract

Optimal peak inspiratory flow rate (PIFR) is crucial for inhalation therapy in patients with chronic obstructive pulmonary disease (COPD). However, little is known about the impact of PIFR-guided inhalation therapy on the clinical outcomes among patients with varying severities of COPD. A PIFR-guided inhalation therapy, including PIFR assessment and PIFR-guided inhaler education, was introduced in a pay-for-performance COPD management program in National Taiwan University Hospital. Among 383 COPD patients, there was significant reduction in incidence of severe acute exacerbation in the PIFR-guided inhalation therapy (PIFR group) than conventional inhaler education (control group) (11.9 vs. 21.1%, *p* = 0.019) during one-year follow-up. A multivariable Cox’s proportional-hazards analysis revealed that the PIFR-guided inhalation therapy was a significant, independent factor associated with the reduced risk of severe exacerbation (adjusted hazard ratio = 0.49, 95% confidence interval, 0.28–0.84, *p* = 0.011). Subgroup analysis found PIFR-guided inhalation therapy was more beneficial to patients with older age, short body stature, COPD stage 1&2, group C&D (frequent exacerbation phenotype), and using multiple inhalers. This study showed the PIFR-guided inhalation therapy significantly reduced the incidence of severe acute exacerbation than conventional inhaler education in patients with COPD. Careful PIFR-assessment and education would be crucial in the management of COPD.

## Introduction

Inhalational drug administration and inhaled therapeutic agents play a major role in the pharmacological management of chronic obstructive pulmonary disease (COPD) (Global Initiative for Chronic Obstructive Lung Disease, 2020; Celli and Wedzicha, 2019). A patient’s ability or skill pertaining to the proper use of inhalers and good compliance are both critical factors associated with the attainment of adequate disease control (Molimard et al., 2017; Gregoriano et al., 2018). Previous literature has elaborately discussed the technique errors during inhalation therapy (Laube et al., 2011; Usmani et al., 2018; Cho-Reyes et al., 2019; Usmani, 2019), but little has mentioned the impact of improper peak inspiratory flow rate (PIFR) in clinical outcomes (Molimard et al., 2017; Gregoriano et al., 2018; Usmani et al., 2018).

Either inadequate or excessive inspiratory flow rate during inhalation therapy has negative impact on drug delivery (Usmani et al., 2005; Laube et al., 2011; Ibrahim et al., 2015). Inadequate inspiratory flow rate can lead to inappropriate deagglomeration of the drug into small particles (Laube et al., 2011), while excessive inspiratory flow rate leads to increased drug deposition in the oropharyngeal regions and reduced deposition in the peripheral regions of the lung (Usmani et al., 2005; Ibrahim et al., 2015). Several previous studies not only have showed high prevalence of improper PIFR among COPD patients but also emphasized the importance of regular assessment of PIFR (Janssens et al., 2008; Malmberg et al., 2010; Mahler et al., 2013; Kawamatawong et al., 2017; Mahler, 2017; Ghosh et al., 2019; Chen et al., 2020; Harb et al., 2020).

Based on PIFR values, clinicians can instruct patients to inhale medication with precise inhalation force or prescribe inhaler with proper resistance. Nevertheless, the impact of PIFR-guided inhalation therapy on COPD patients has not been evaluated. We hypothesized that COPD patients would benefit from PIFR-guided inhalation therapy and have improved clinical outcome. This study was thus conducted to investigate the impact of PIFR-guided inhalation therapy on the incidence of exacerbation and all-cause mortality in COPD patients with continuous inhalation therapy.

## Materials and Methods

### Study Design

In Taiwan, a pay-for-performance program for COPD patients was introduced by the National Health Insurance Administration since 2017, which involved the prospective documentation of patient’s evaluation results, medication prescriptions and the episodes of disease exacerbation. The diagnosis of COPD was made in accordance with the Global Initiative for Chronic Obstructive Lung Disease (GOLD) criteria that is defined as follows: post-bronchodilator forced expiratory volume in 1 s (FEV1) to forced vital capacity (FVC) ratio (FEV1/FVC ratio) of less than 70%. The inhalers were prescribed by attending physicians and all patients received inhaler education from the same case manager. The inhaler education included demonstration of the steps of inhaler handling and subsequent practices under direct observation at intervals of one to three months.

### PIFR-Guided Inhalation Therapy

**FIGURE 1:**
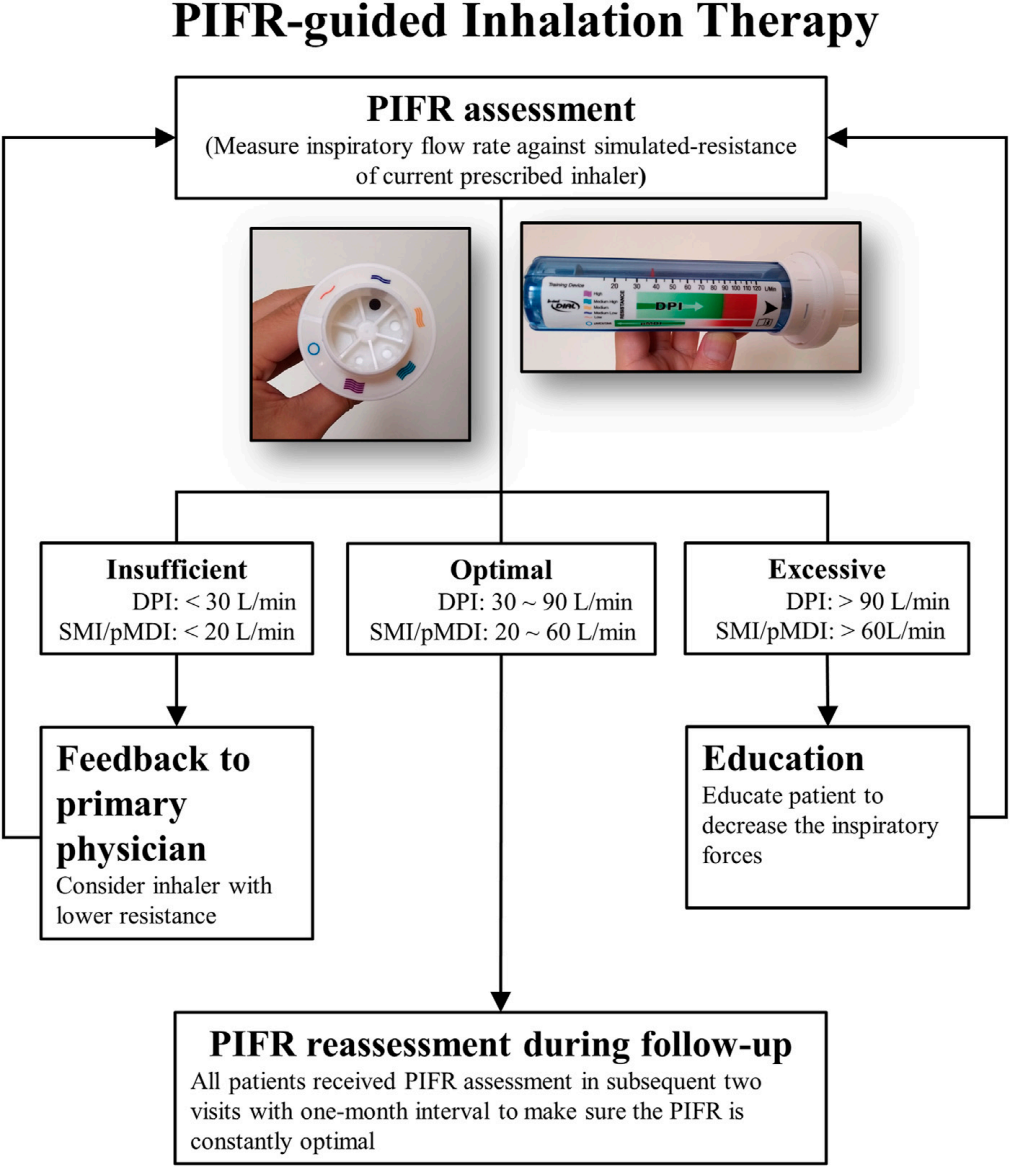
Peak Inspiratory Flow Rate guided inhalation therapy; DPI: dry powder inhaler; SMI: soft mist inhaler; pMDI: pressurized metered dose inhaler; PIFR: peak inspiratory flow rate.

The PIFR-guided inhalation therapy, including PIFR assessment and PIFR-guided inhaler education, was introduced since June 2018 (Figure 1). The In-Check Dial G16 (Clement-Clarke International Ltd., Harlow, United Kingdom) was used to measure PIFR under simulated-resistance of the prescribed inhaler. Patients, in sitting position, were asked to perform three inspiratory maneuvers through In-Check Dial G16. The optimal PIFR for dry powder inhalers (DPI) ranges from 30 to 90 L/min and 20–60 L/min for a soft mist inhaler (SMI) or a pressurized metered dose inhaler (pMDI) (Laube et al., 2011; Mahler, 2017; Sanders, 2017). If the highest PIFR observed was insufficient for prescribed inhaler, the PIFR against lower resistance was measured. The PIFR results was given as a feedback to the primary physicians immediately for reconsideration of current description. If the PIFR observed was excessive, the patient will be taught to decrease the inspiratory forces. With direct biofeedback from In-Check Dial G16, patients can easily learn how to use the appropriate inhalation strength. Patients were required to return to the case manager for reassessment of PIFR and repeatedly assessed during two subsequent clinical visits to make sure its optimality.

### Data Collection and Follow-Up

**FIGURE 2:**
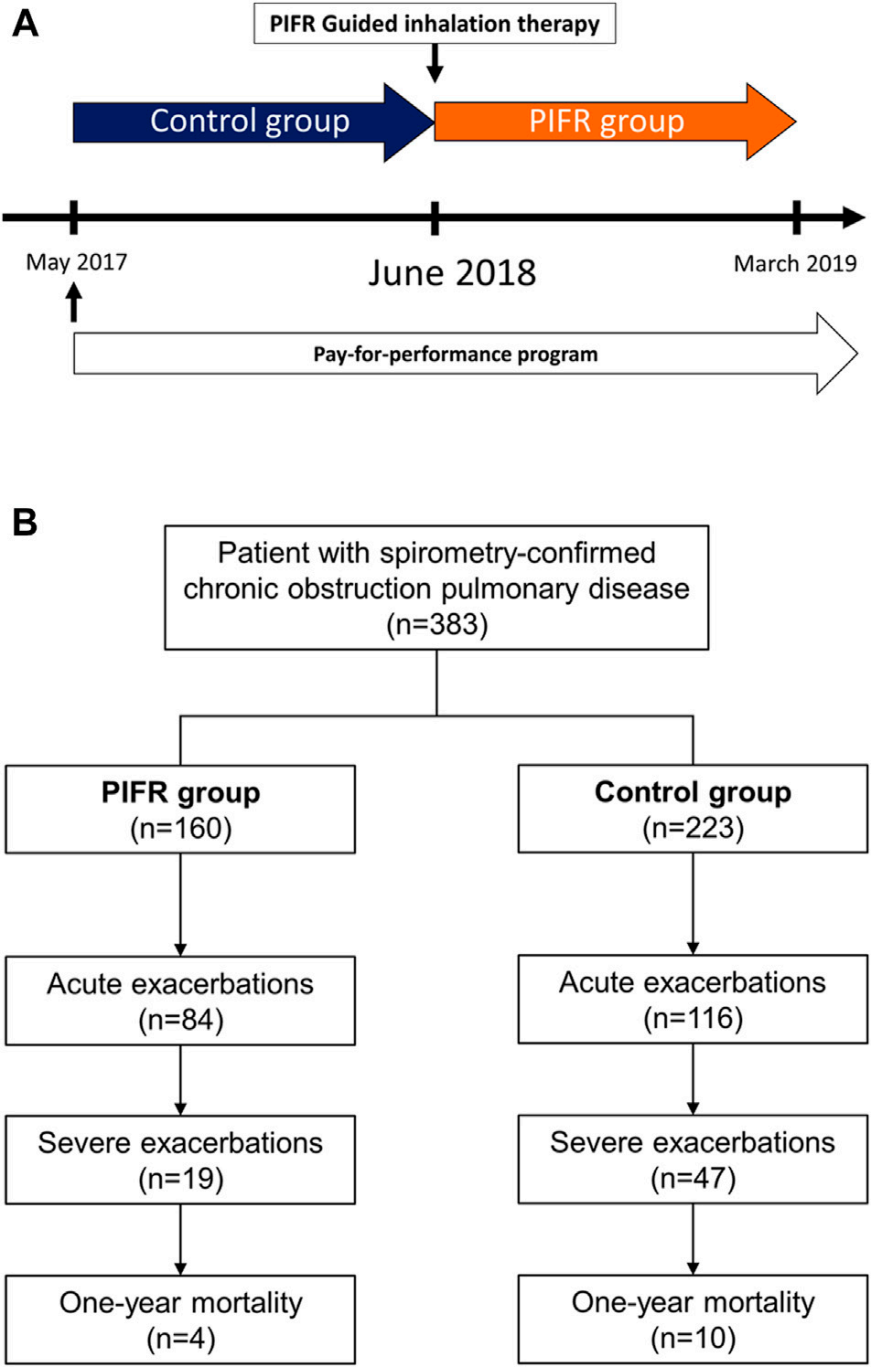
**(A)** A mirror image study design comparing patients with COPD before and after the introduction of peak inspiratory flow rate guided inhalation therapy. **(B)** Flow chart depicting the enrollment. PIFR group: patients who received PIFR-guided inhalation therapy.

Figure 2 showed during May 2017 to March 2019, all COPD patients in pay-for-performance program in National Taiwan University Hospital were enrolled. A mirror image study design was employed to access the impact of PIFR-guided inhalation therapy. Patients who received conventional inhaler education before PIFR-guided therapy introduction was defined as control group and after the intervention, subjects who received PIFR-guided inhalation therapy was defined as PIFR group (Figure 2).

The patients demographics, results of pulmonary function tests, smoking status, severity of COPD (in accordance with the GOLD criteria), type of inhaler devices used, the inhaled medications and vaccination were documented. Acute exacerbations were classified into three categories, in accordance with the GOLD report, namely, mild (relieved by short acting bronchodilators), moderate (treatment involves antibiotics or oral corticosteroids, in addition to short acting bronchodilators) and severe (involves visits to the emergency department or situations that require hospitalization). Data pertaining to the timing of the occurrence of mild exacerbations were self-reported, whereas the dates pertaining to the episodes of moderate or severe exacerbations were gathered from the medical records. In addition, the time interval between the initial inhaler education and the first episode of disease exacerbation were analyzed. This study was approved by the institutional review board of the National Taiwan University Hospital (201905058RINB).

### Statistical Analysis

Categorical variables were compared using the chi-square test or Fisher’s exact test, as appropriate. Difference in continuous variables were analyzed by means of the Mann-Whitney U test. The data are presented as numbers (percentages) and median (25th, 75th percentile). The present study considered a two-sided *p*-value of 0.05 as statistically significant. The annual incidence rate of acute exacerbation was calculated as event/patient/year and presented with mean (95% confidence intervals). The incidence rates in each group were compared with incidence rate ratio and the statistics of significance were determined using negative binominal statistical test. Kaplan-Meier curve with log-rank test was used to analyze the time interval from inhaler education to the first episode of exacerbation. Univariate and multivariate analysis were performed based on factors significantly differed between both groups in baseline characteristics and on possible confounding factors mentioned in previous literatures (Global; Malmberg et al., 2010; Mahler et al., 2013; Celli and Wedzicha, 2019; Ghosh et al., 2019; Chen et al., 2020). The median value of age and height were used as the cutting point in subgroup analysis. The statistical analyses were performed using the STATA version 14 software (StataCorp LLC, TX).

## Results

### Baseline Characteristics

A total of 383 patients with COPD were enrolled. Among them, 223 (58.2%) patients received conventional inhaler education (control group) and, after introduction of PIFR-guided inhalation therapy, 160 (41.8%) patients received PIFR-guided inhalation therapy (PIFR group). As shown in Table 1, the baseline characteristics including the age, height, weight, results of pulmonary function tests, severity of the disease, smoking status, types of inhalers used, inhaled medications and vaccination status were similar.

**TABLE 1:** Baseline characteristics of the study subjects.

	Total (*n*=383)	PIFR group (*n*=160)	Control group (*n*=223)	*p*-value
Age, year	73.4 (65.8,78.7)	73.3 (66.8,78.7)	73.4 (65.6,78.8)	0.798
Male	351 (91.6)	153 (95.6)	198 (88.8)	0.017
Weight, kg	63 (55.0,70.0)	63 (56.0,71.0)	62 (53.7,69.0)	0.175
Height, cm	163.1 (159.3,168.0)	164 (159.3,168.0)	163 (159.3,167.4)	0.322
Body mass index, kg/m^2^	23.1 (20.5,25.7)	23.1 (20.6,25.8)	23 (20.5,25.7)	0.569
FEV1, L	1.48 (1.19,1.86)	1.50 (1.24,1.93)	1.46 (1.15,1.80)	0.144
FEV1, % predicted	70.5 (56.5,83.4)	71.9 (57.4,85.2)	69.9 (56.5,82.6)	0.523
Maximal inspiratory pressures, cmH_2_O	−75.6 (−55.0,−95.0)	−75.3 (−55.0,−93.0)	−75.9 (−57.0,−95.0)	0.877
COPD GOLD group				0.748
GOLD group A	102 (26.6)	44 (27.5)	58 (26.0)	
GOLD group B	203 (53.0)	85 (53.1)	118 (52.9)	
GOLD group C	18 (4.7)	9 (5.6)	9 (4.0)	
GOLD group D	60 (15.7)	22 (13.8)	38 (17.0)	
Current smoker	106 (27.7)	49 (30.6)	57 (25.6)	0.512
Inhaler type				0.556
pMDI	12 (3.1)	5 (3.1)	7 (3.1)	
SMI	62 (16.2)	24 (15.0)	38 (17.0)	
DPI	229 (59.8)	94 (58.8)	135 (60.6)	
DPI+ (pMDI or SMI)	60 (15.7)	31 (19.4)	29 (13.0)	
SMI+pMDI	16 (4.2)	4 (2.5)	12 (5.4)	
DPI+DPI	4 (1.0)	2 (1.2)	2 (0.9)	
Medication				0.399
LAMA	94 (24.5)	41 (25.6)	53 (23.8)	
LABA	12 (3.1)	7 (4.4)	5 (2.2)	
LAMA+LABA	148 (38.6)	55 (34.4)	93 (41.7)	
ICS+LABA	54 (14.1)	20 (12.5)	34 (15.2)	
ICS+LAMA	1 (0.4)	0 (0.0)	1 (0.5)	
ICS+LAMA+LABA	74 (19.3)	37 (23.1)	37 (16.6)	
Vaccination				
Influenza	260 (67.9)	117 (73.1)	143 (64.1)	0.063
Pneumococcus	241 (62.9)	105 (65.6)	136 (61.0)	0.354

Data presented as n (%) or median (25th, 75th percentile). FEV1: forced expiratory volume in 1 s; GOLD: global initiative for chronic obstructive lung disease; pMDI: pressurised metered dose inhaler; SMI: soft mist inhaler; DPI: dry powder inhaler; LAMA: long-acting muscarinic antagonist; LABA: long-acting beta2 agonist; ICS: inhaled corticosteroid; PIFR: peak inspiratory flow rate. PIFR group: patients who received PIFR-guided inhalation therapy.

Among 160 patients in PIFR group, 79 (49.4%) patients had inappropriate initial PIFR. In those who use dry powder inhalers (DPI), 9 (16%) out of fifty-seven patients displayed inadequate PIFR. After the feedback to primary physician, the inhaler was substituted with another type of inhaler in six patients. On the contrary, 32 (56%) DPI users, 18 (90%) pMDI users and 26 (87%) SMI users displayed excessive PIFR, and after education, they performed optimal PIFR in the subsequent check-ups.

### Severe Acute Exacerbation

**FIGURE 3:**
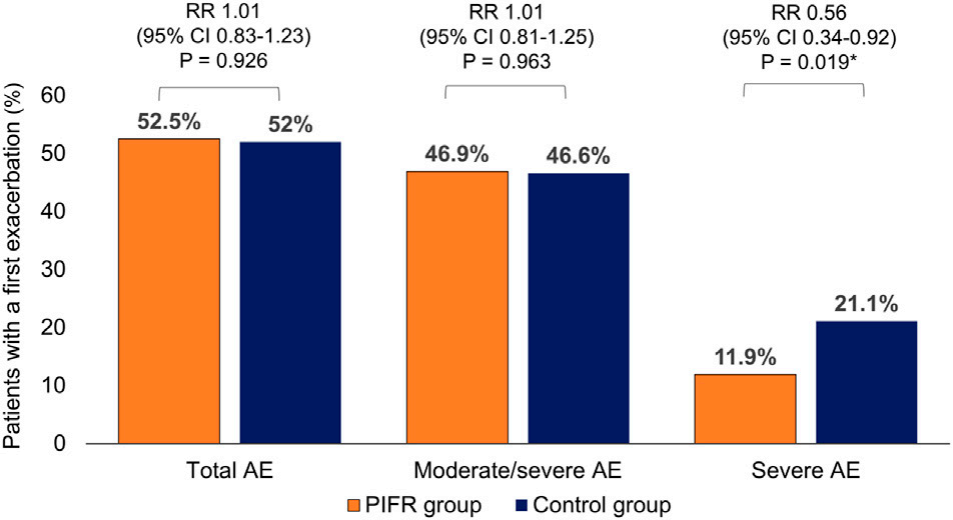
Incidence of acute exacerbation of chronic obstructive pulmonary disease within one year among the patients in the PIFR group and control group. AE: acute exacerbation. RR: relative risk. CI: confidence intervals.

**FIGURE 4:**
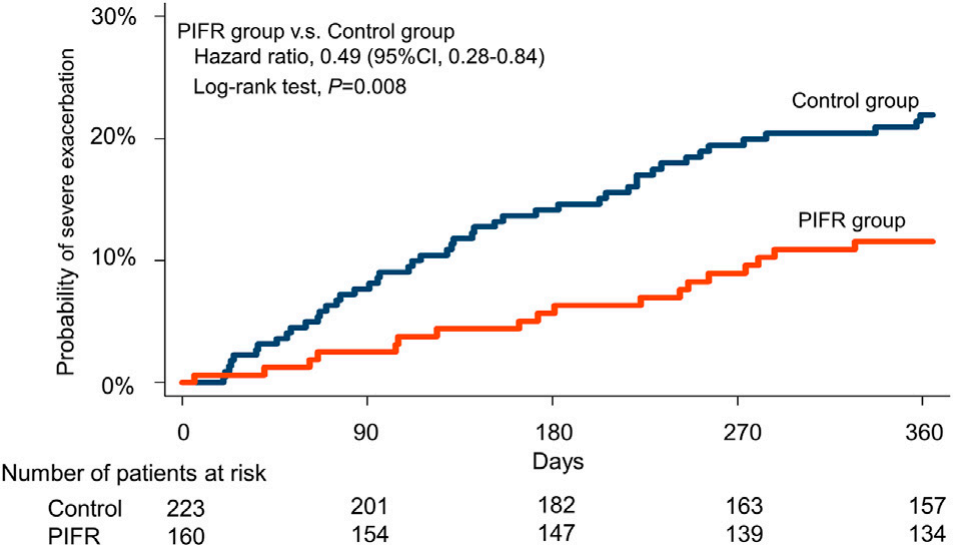
Kaplan-Meier time-to-event plot and log-rank test for time to first severe acute exacerbation among PIFR group and control group.

Compared to the control group, there was a significant reduction in the incidence (11.9 vs. 21.1%, relative risk 0.56, *p* < 0.05, Figure 3) and incidence rate (0.18 vs. 0.43, incidence rate ratio 0.52, *p* < 0.05, Table 2) of severe exacerbation within one-year follow-up in the PIFR group (Hazard ratio 0.49, 95% CI 0.28–0.84, *p* < 0.05, Figure 4). The incidence of total exacerbation and all-cause mortalities were similar among two groups (Supplementary Table S1).

**TABLE 2:** One year incidence rates and incidence rate ratios of acute exacerbation in respect of severity among the study subjects.

	PIFR group (*n* = 160)	Control group (*n* = 223)	Incidence rate ratio (PIFR group to control group)	*p* value
Total AE	0.88 (0.71–1.06)	1.14 (0.94–1.34)	0.84 (0.62–1.13)	0.245
Mild AE	0.11 (0.06–0.16)	0.17 (0.11–0.23)	0.68 (0.37–1.23)	0.203
Moderate AE	0.55 (0.42–0.68)	0.55 (0.28–0.57)	1.11 (0.79–1.57)	0.546
Severe AE	0.18 (0.09–0.27)	0.43 (0.28–0.57)	0.52 (0.32–0.87)	0.012
Moderate-severe AE	0.77 (0.60–0.94)	0.98 (0.79–1.16)	0.88 (0.64–1.20)	0.401

Data presented as mean (95% confidence intervals). AE: acute exacerbation. Incidence rate was calculated as events/patient/year. PIFR group: patients who received PIFR-guided inhalation therapy.

In univariable analysis using Cox’s proportional-hazards analysis, the introduce of PIFR guided inhalation therapy, higher BMI, FEV1 ≥ 50% (GOLD stage1&2), less exacerbation in previous one year (GOLD group AandB) and single inhaler usage were associated with reduced probability of severe acute exacerbation. Multivariable analysis showed that the introduce of PIFR-guided inhalation therapy (Hazard ratio, HR = 0.48, 95% confidence interval, CI = 0.28–0.83, *p* < 0.05) and less exacerbation in previous one year (GOLD group AandB classification) (HR = 0.34, 95% CI = 0.21–0.57, *p* < 0.05) were two independent factors associated with the lower risk of severe acute exacerbation within one year (Table 3).

**TABLE 3:** Factors associated with the risk of severe exacerbation within one year.

	Univariable analysis			Multivariable analysis		
	Hazard ratio	95% CI	*p*-value	Hazard ratio	95% CI	*p*-value
PIFR vs. control group	0.49	0.28–0.84	0.009	0.48	0.28–0.83	0.008
Age, year	1.01	0.98–1.03	0.626	—	—	—
Male gender	0.90	0.39–2.09	0.811	—	—	—
BMI, kg/m^2^	0.93	0.87–0.99	0.046	0.96	0.90–1.03	0.239
GOLD stage (1&2 vs. 3&4)	0.43	0.26–0.73	0.002	0.58	0.32–1.04	0.069
GOLD group (A&B vs. C&D)	0.31	0.19–0.51	<0.001	0.34	0.21–0.57	<0.001
Multiple vs. single inhaler device	1.78	1.05–3.02	0.031	1.46	0.83–2.57	0.194
ICS vs. no ICS	1.457	0.89–2.39	0.135	—	—	—
Current smoker vs. quitted	0.79	0.44–1.40	0.417	—	—	—
Vaccination						
Influenza	0.87	0.52–1.46	0.603	—	—	—
Pneumococcus	1.33	0.79–2.25	0.288	—	—	—

PIFR; peak inspiratory flow rate; PIFR group: patients who received PIFR-guided inhalation therapy; BMI: body mass index; GOLD: global initiative for chronic obstructive lung disease; ICS: inhaled corticosteroid; CI: confidence interval.

**FIGURE 5:**
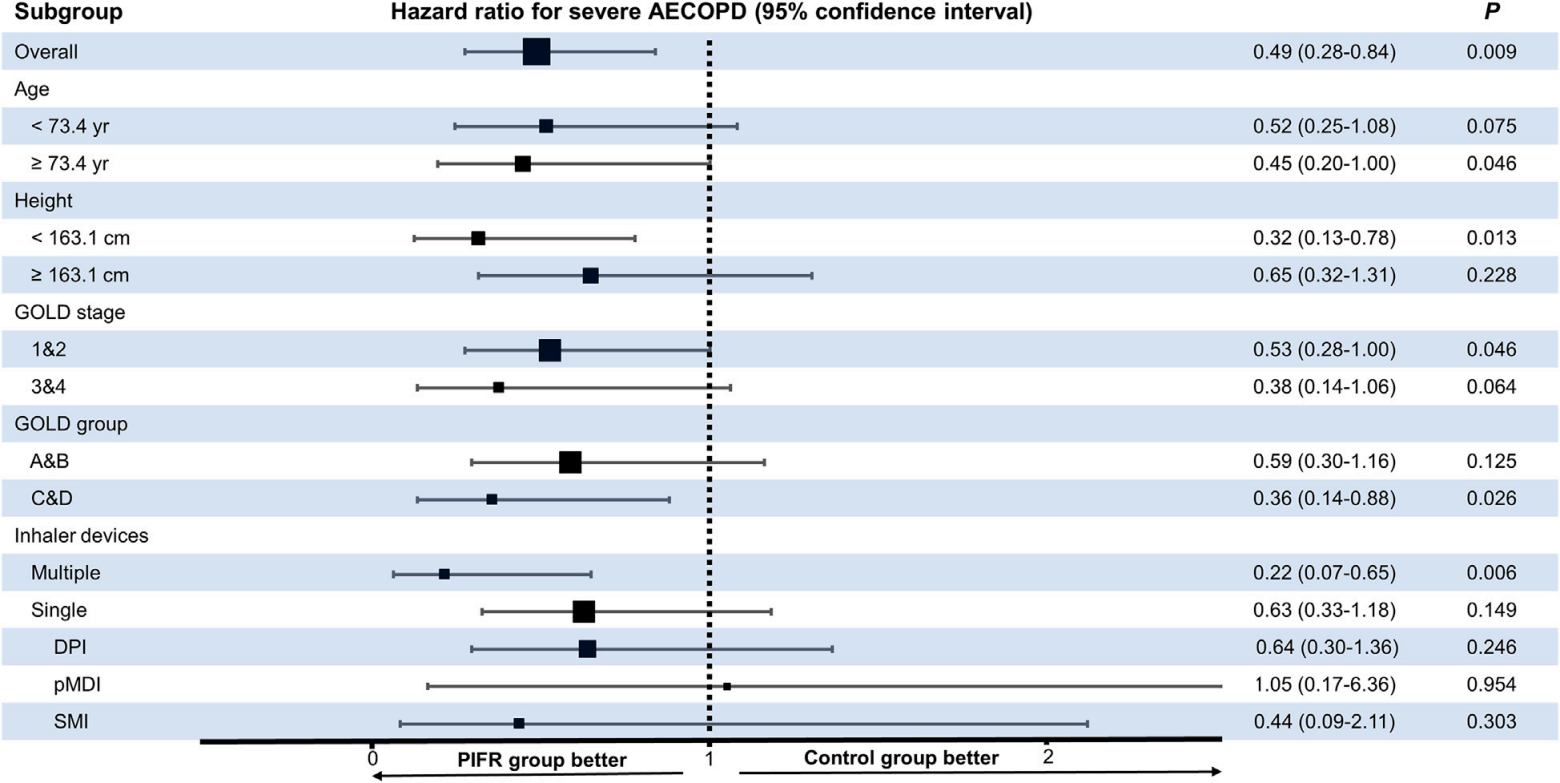
Forrest plot pertaining to the hazard ratios of the time interval to the first episode of severe acute exacerbation of chronic obstructive pulmonary disease (AECOPD) among the patients in the PIFR and control groups, stratified by subgroups. GOLD: Global Initiative for Chronic Obstructive Lung Disease; pMDI: pressurized metered dose inhaler; SMI: soft mist inhaler; DPI: dry powder inhaler.

Figure 5, Supplementary Figure S1 showed PIFR-guided inhalation therapy reduced risk of severe acute exacerbation in one year especially in subgroups of older age (HR = 0.45, 95% confidence interval, CI = 0.20–1.00, *p* < 0.05), shorter body stature (HR = 0.32, 95% confidence interval, CI = 0.13–0.78, *p* < 0.05), COPD GOLD stage 1&2 (HR = 0.53, 95% confidence interval, CI = 0.28–1.00, *p* = 0.05), COPD GOLD group C&D (HR = 0.36, 95% confidence interval, CI = 0.14–0.88, *p* < 0.05), and using at least two types of inhalers at the same time (HR = 0.22, 95% confidence interval, CI = 0.07–0.65, *p* < 0.05).

## Discussion

In this retrospective analysis of prospectively collected data, we found COPD patients receiving PIFR-guided inhalation therapy have lower probability of experiencing severe acute exacerbation. The PIFR-guided inhalation therapy is especially beneficial to patients of older age, short body stature, COPD GOLD stage 1&2 (FEV1 ≥ 50% predicted of value), COPD GOLD group C&D (frequent exacerbation phenotype), and using multiple inhalers at the same time. To our knowledge, no study has been undertaken to evaluate the impact of PIFR-guided inhalation therapy during COPD treatment, making this the first.

The devices handling errors during inhalation therapy has been known as important risk factors that influence the occurrence of exacerbation of COPD (Global Initiative for Chronic Obstructive Lung Disease, 2020; Molimard et al., 2017; Usmani et al., 2018). High prevalence of inappropriate PIFR has also been found in patients using DPIs (Janssens et al., 2008; Mahler, 2017; Ghosh et al., 2019; Chen et al., 2020; Harb et al., 2020) and several previous studies showed 20–78% study population had suboptimal PIFR. Furthermore, in our previous study, a considerable proportion of COPD patients had excessive PIFR when using medium-low or low resistance DPIs (Chen et al., 2020). Because excessive PIFR may lead to increased oropharyngeal deposition and suboptimal PIFR results in improper delivery of the medication, inappropriate PIFR could result in poor disease control and increasing frequency of disease exacerbation. This concept was previously demonstrated by the two randomized control trials conducted by Mahler and colleagues in 2014 and 2019 (Mahler et al., 2014; Mahler et al., 2019). The first trial compared the efficacy of nebulized short acting beta agonist (SABA) with SABA administered through Diskus inhaler. Among twenty patients with suboptimal PIFR, authors found that nebulized SABA achieved better improvement in FVC and inspiratory capacity, compared to the SABA administered through the Diskus inhaler (Mahler et al., 2014). Another phase 3b study reported by the same authors involved 206 patients with suboptimal PIFR and found the treatment using nebulized long acting muscarinic antagonist in the patients with FEV1 below 50% of the predicted value resulted in greater incremental increase in FEV1, compared to using HandiHaler inhaler (Mahler et al., 2019). Based on the above two studies, those who use dry powder inhalers with suboptimal PIFR were referred back to primary physician for reconsideration of device choice in this study. The resolution is usually choosing a DPI with lower resistance or changing to pMDI or SMI.

In addition, improper PIFR is also a point of concern in using SMI and pMDI, particularly in untrained patients (Haidl et al., 2016). A cross-sectional study by Gregoriano and colleagues, which involved 165 patients with COPD and asthma, reported that 17% of the patients did not perform the proper inhalation maneuver (Gregoriano et al., 2018). A study by Peter and colleagues screened 13 COPD patients and reported that all the patients inhaled too fast with pMDI, prior to the initiation of training (Brand et al., 2008). After the inhaler handling training involving PIFR assessment and breath-holding techniques, the mean whole lung deposition increased from 37 to 53% of the delivered dose, whereas the oropharyngeal deposition decreased from 56 to 45% of the delivered dose. In this study, we found approximately 87–90% of patients using pMDI and SMI have excessive PIFR. The problem could be solved by teaching patients to inhale gently and slowly with the direct visual feedback of PIFR values on the In-Check Dial G16 to patients. This is among the core concepts of PIFR-guided inhalation therapy which involve not only identifying suboptimal PIFR but also helping patients to adjust themselves to exerting the ideal effort on medication inhalation.

A study by Loh and colleagues showed among 123 patients, 64 (52%) had suboptimal PIFR and had greater scores in the COPD assessment test, more frequent re-admissions due to COPD within 90 days, and fewer days to re-admission after treatment initiation (Loh et al., 2017). In this study, we also found among 160 patients having PIFR assessment, 79 (49.4%) patients had inappropriate initial PIFR. After PIFR guided inhalation therapy, there was a significant reduction in the incidence and risk of severe acute exacerbation. Further, since the PIFR guided inhalation therapy is an independent factor associated with reduced probability of severe acute exacerbation both in univariable and multivariable analysis models, the importance of this intervention cannot be over emphasized.

According to previous reports, several factors such as older age, female gender, short stature, reduced inspiratory capacity, inspiratory muscle weakness and acute status post exacerbation were strongly associated with suboptimal PIFR (Weiner and Weiner, 2006; Janssens et al., 2008; Malmberg et al., 2010; Mahler et al., 2013; Kawamatawong et al., 2017; Mahler, 2017; Sharma et al., 2017; Ghosh et al., 2019; Harb et al., 2020; Samarghandi et al., 2020). It can explain why in subgroup analysis of this study patients with elder age, short body stature and frequent exacerbation phenotype (GOLD group C&D) had reduced risk of severe acute exacerbation after receiving PIFR-guided inhalation therapy (Figure 5, Supplementary Figure S1).

Furthermore, the PIFR-guided inhalation therapy may be particularly important in patients prescribed with a combination of different types of devices, such as DPI with SMI, DPI with pMDI or even two types of DPIs with different resistances. A previous study by Hira and colleagues analyzed the inhalation flow pattern in ten participants who were prescribed with a combination of DPI and SMI (Hira et al., 2018), they found the patients frequently confused DPI with SMI. In the current study, we also found more patients using multiple inhalers at one time in those with initial inappropriate PIFR than those with initial appropriate PIFR (30.4 vs. 8.6%, *p* = 0.001, Supplementary Table S1). After PIFR assessment and inhaler education, significant improvement in the inhalation profile was observed. This could explain why the implementation of PIFR-guided inhalation therapy was especially beneficial to patients using multiple inhaler devices (Figure 5).

This study has certain limitations. Firstly, the predominance of the male gender, which is a feature of the population affected by COPD in Taiwan, may not represent the spectrum of the global population affected. Accordingly, several previous studies have reported that the female gender is an important predictor of reduced PIFR. In contrast, it was not observed to have significant effect in this study. Second, the data pertaining to the events of acute exacerbation in some patients may have been overlooked and were not included in the current retrospective analysis, especially the events that occurred in other hospitals. However, because all the patients were prospectively enrolled in the national COPD care program of Taiwan, most data pertaining to the relevant events and other medical details can be traced on the e-cloud system of National Health Insurance in Taiwan. Finally, without randomization and blindness, the present study may include confounding factors, such as the implementation of pulmonary rehabilitation (Weiner and Weiner, 2006), which may affect the inspiratory flow rate; the change of therapeutic strategies or regimens with disease status or with updated guidelines such as serum biomarker directed inhaled corticosteroid treatment (Sivapalan et al., 2021), better treatment of comorbidities by multidiscipline members, which may all affect the outcome of acute exacerbation. In the future, further research involving prospectively randomized control study is necessary to overcome these limitations.

## Conclusion

Inappropriate PIFR during inhalation therapy is indeed an important factor in inhaler handling, thus the role of PIFR assessment had been put on emphasis in recent years and even proposed to be an emerging biomarker in COPD treatment (Mahler, 2019). In this cohort study, we demonstrated that the incorporation of PIFR-guided inhalation therapy into COPD treatment plan could reduce the risk of severe acute exacerbation. It is especially beneficial in patients with older age, short body stature, FEV1 ≥ 50%, frequent exacerbation phenotype and using multiple inhalers at the same time.

## Data Availability

The raw data supporting the conclusions of this article will be made available by the authors, without undue reservation.
