# Supplementary material for: Peak-Inspiratory-Flow-Rate Guided Inhalation Therapy Reduce Severe Exacerbation of COPD
Source: Front Pharmacol. 2021 Jun 29;12:704316. doi: 10.3389/fphar.2021.704316 (PMC8277232; doi:10.3389/fphar.2021.704316)
Supplement: Supplementary file 1 [file DataSheet1.docx]

Supplementary Material

**Supplementary Table 1**: Distribution of the episodes of acute exacerbations and mortality among the study subjects.

|  | PIFR group  (n=160) | Control group  (n=223) | p-value |
| --- | --- | --- | --- |
| Acute exacerbations |  |  |  |
| Total |  |  | 0.046 |
| 1 event/year | 52 (32.5) | 51 (22.9) |  |
| 2 events/year | 18 (11.3) | 27 (12.1) |  |
| ≥3 events/year | 14 (8.8) | 38 (17.0) |  |
| Mild |  |  | 0.188 |
| 1 event/year | 16 (10.0) | 21 (9.4) |  |
| 2 events/year | 1 (0.6) | 8 (3.6) |  |
| Moderate |  |  | 0.434 |
| 1 event/year | 41 (25.6) | 43 (19.3) |  |
| 2 events/year | 10 (6.3) | 19 (8.5) |  |
| ≥3 events/year | 9 (5.6) | 11 (4.9) |  |
| Severe |  |  | 0.037 |
| 1 event/year | 14 (8.8) | 23 (10.3) |  |
| 2 events/year | 3 (1.9) | 14 (6.3) |  |
| ≥3 event/year | 2 (1.3) | 10 (4.5) |  |
|  |  |  |  |
| Mortality |  |  |  |
| 90-day mortality | 0 (0) | 4 (1.8) | 0.143 |
| One-year mortality | 4 (2.5) | 10 (4.5) | 0.412 |

Data presented as n (%). PIFR: peak inspiratory flow rate. PIFR group: patients who received PIFR-guided inhalation therapy.

**Supplementary Table 2**: Baseline characteristics of the subgroups in the PIFR group according to initial PIFR condition (Optimal versus Inappropriate)

|  | Optimal baseline PIFR (n=81) | Inappropriate baseline PIFR (n=79) | p-value |
| --- | --- | --- | --- |
| Age, year | 73.9(66.4,78.6) | 72.7(67.2,78.7) | 0.957 |
| Male, n (%) | 75 (92.6) | 78 (98.7) | 0.117 |
| Weight, kg | 62(55.0,69.3) | 64(56.8,71.0) | 0.238 |
| Height, cm | 163.7(159.0,168.0) | 164(160.6,168.0) | 0.432 |
| Body mass index, kg/m^2^ | 22.9(20.7,25.6) | 23.7(20.5,25.8) | 0.447 |
| FEV1, L | 1.37(1.15,1.79) | 1.58(1.29,1.97) | 0.033 |
| FEV1, % predicted | 70(55.0,83.0) | 72.5(60.5,88.2) | 0.321 |
| FVC, L | 2.65(2.37,3.27) | 2.93(2.43,3.40) | 0.219 |
| FVC, % predicted | 97.6(86.8,115.4) | 99(85.6,112.1) | 0.939 |
| GOLD group |  |  | 0.737 |
| GOLD group A | 20 (24.7) | 24 (30.4) |  |
| GOLD group B | 46 (56.8) | 39 (49.4) |  |
| GOLD group C | 5 (6.2) | 4(5.1) |  |
| GOLD group D | 10 (12.3) | 12 (15.1) |  |
| Current smoker | 27 (33.3) | 22 (27.9) | 0.495 |
| Inhaler type |  |  | <0.001 |
| pMDI (other than Berotec) | 3 (3.7) | 2 (2.5) |  |
| SMI | 7 (8.6) | 17 (21.5) |  |
| DPI | 63 (77.8) | 33 (41.8) |  |
| DPI+pMDI or DPI+SMI | 7 (8.6) | 24 (30.4) |  |
| pMDI+SMI | 1 (1.3) | 3 (3.8) |  |

Data presented as n (%) or median (25^th^, 75^th^ percentile). FEV1: forced expiratory volume in one second; FVC: forced vital capacity; GOLD: Global Initiative for Chronic Obstructive; pMDI: pressurised metered dose inhaler; SMI: soft mist inhaler; DPI: dry powder inhaler; AE: acute exacerbation; PIFR: peak inspiratory flow rate. PIFR group: patients who received PIFR-guided inhalation therapy.


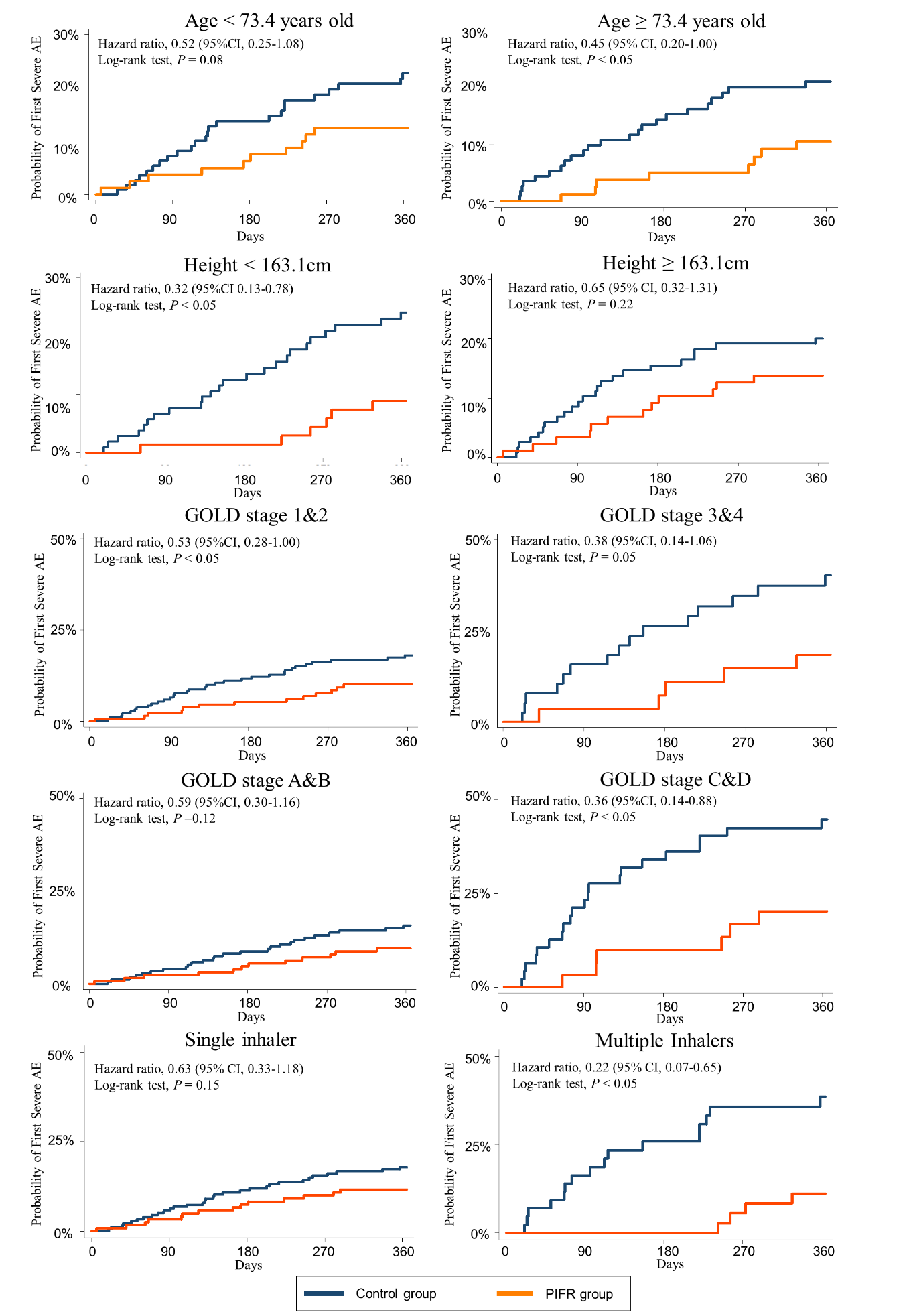
**Supplementary Figure 1** Kaplan-Meier time-to-event plot and log-rank test pertaining to the time interval to the first episode of severe acute exacerbation of chronic obstructive pulmonary disease (severe AECOPD) in the PIFR group and the control group stratified by age, height, GOLD stage, GOLD group and inhaler types.
